# Supplementary material for: SNP-by-CpG Site Interactions in ABCA7 Are Associated with Cognition in Older African Americans
Source: Genes (Basel). 2022 Nov 18;13(11):2150. doi: 10.3390/genes13112150 (PMC9691156; doi:10.3390/genes13112150)
Supplement: Supplementary file 1 [file genes-13-02150-s001.zip › genes-1938107-supplementary.pdf]

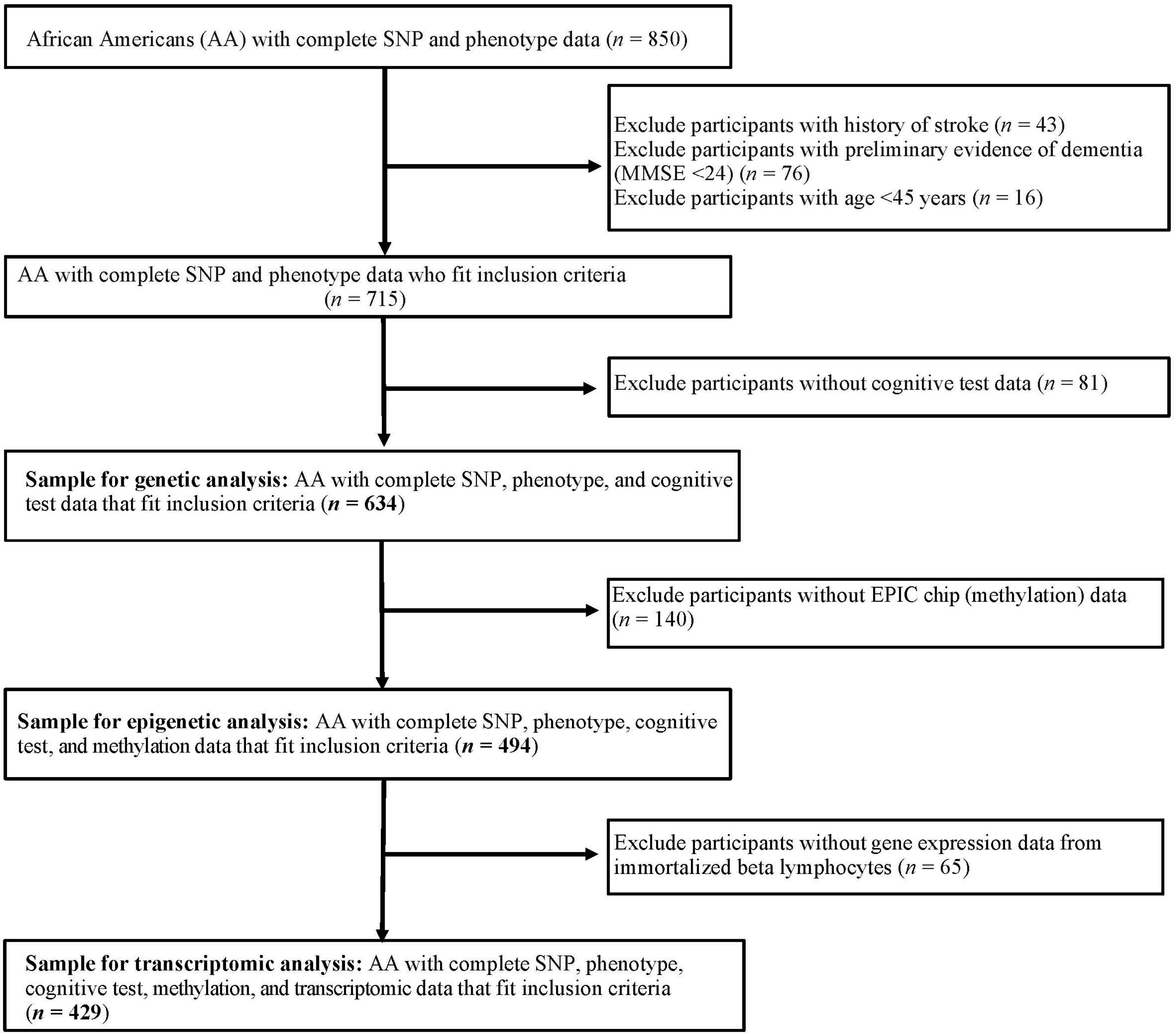

Figure S1: Flow diagram illustrating sample sizes for genetic (*n* = 634), epigenetic (*n* = 494), and transcriptomic (*n* = 429) analyses in GENOA AA.

Genetic associations

Model 1: General cognitive function ~ SNP + age at cognition measurement + sex + PC1-4+ familial relatedness (random effect)

Model 2: Model 1 + educational attainment

Model 3: Model 2 + *APOE ε2 + APOE ε4*

Epigenetic associations

Model 1: General cognitive function ~ CpG site + sex + age at cognition measurement + age difference between methylation and cognition measurements + smoking status + PC1-4 + familial relatedness (random effect)

Model 2: Model 1 + educational attainment

Model 3: Model 2 + *APOE* ε2 + *APOE* ε4

Genetic-epigenetic interaction associations

Model 4: General cognitive function ~ SNP + CpG + SNP*CpG + age at cognition measurement + age difference between methylation and cognition measurements + sex + educational attainment + *APOE ε2 + APOE ε4* + smoking status + PC1-4 + familial relatedness (random effect)

Figure S2: Models used to assess genetic, epigenetic and genetic-epigenetic interaction associations with general cognitive function.

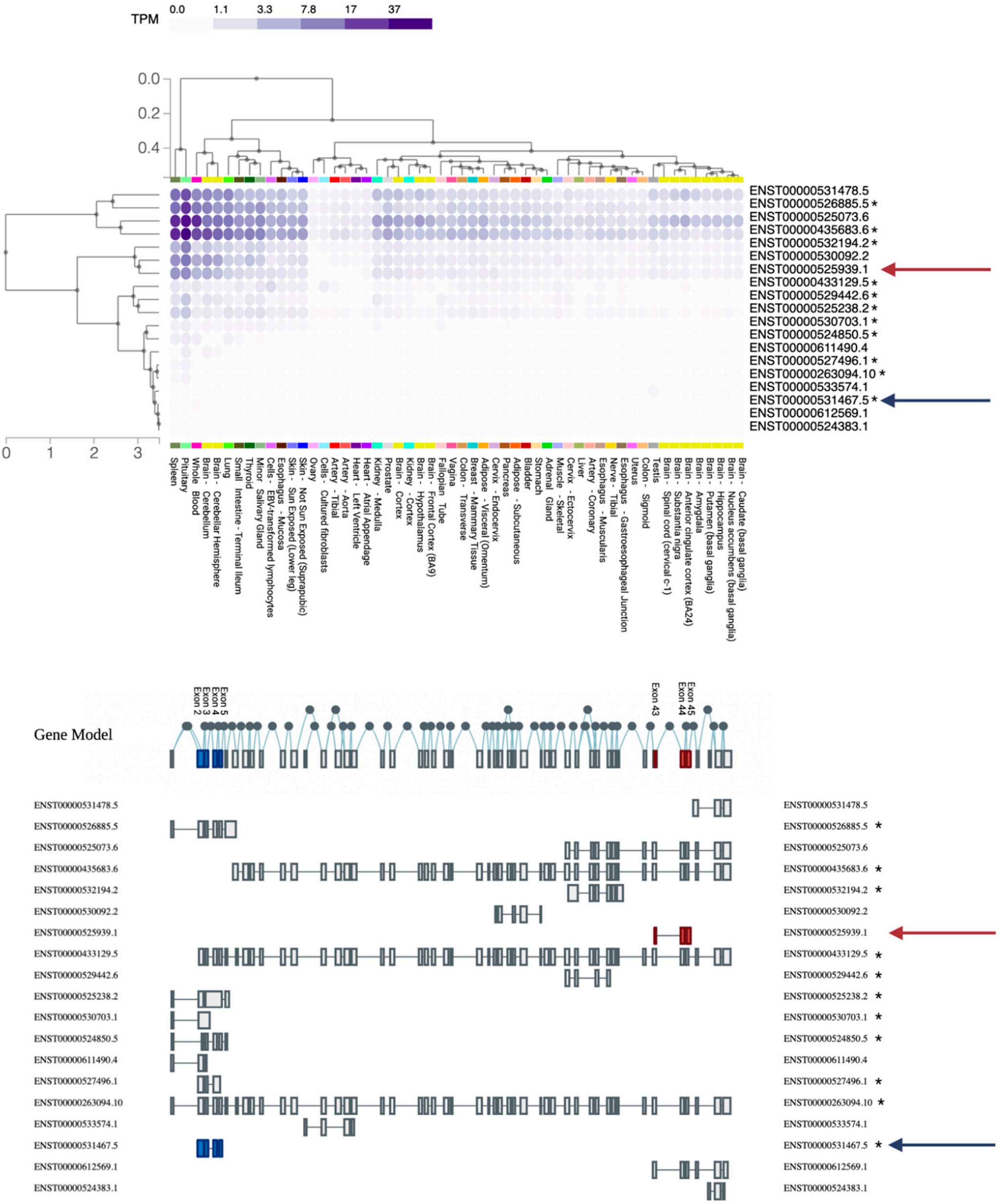

Figure S3: Transcript expression of *ABCA7*: ENSG00000064687 (12 ATP binding cassette subfamily A member 7 [Source: HGNC Symbol; Acc:HGNC:37]). The upper panel shows the tissue expression levels for all *ABCA7* transcripts available in GTEx. The lower panel shows exonic positions of the *ABCA7* transcript isoforms. ENST00000525939 and ENST00000531467, which are associated with rs115550680*cg17316918 and rs3764647*cg22271697 interactions, respectively (Table S10), are indicated by red and blue arrows. Introns within the *ABCA7* gene that are included in each of the two transcripts are colored red and blue correspondingly. Transcripts that are associated with rs115550680 (Table S12) are indicated by asterisks. Figure adapted from https://www.gtexportal.org/home/gene/ENSG00000064687. Data Source: GTEx Analysis Release V8 (dbGaP Accession phs000424.v8.p2) [59].

Table S1. Pearson's correlations among the six cognitive measures (*n* = 634)

	RAVLT	DSST	COWA-FAS	SCWT	TMTA	General cognitive function
RAVLT	1.000					
DSST	0.365***	1.000				
COWA-FAS	0.248***	0.516***	1.000			
SCWT	0.251***	0.516***	0.336***	1.000		
TMTA	0.241***	0.663***	0.419***	0.432***	1.000	
General cognitive function	0.522***	0.874***	0.698***	0.704***	0.791***	1.000

Abbreviations: RAVLT, Rey Auditory Verbal Learning Test; DSST, Digit Symbol Substitution Task; COWA-FAS, Controlled Oral Word Association Test; SCWT, Stroop Color-Word Test; TMTA, Trail Making Test A

***p<0.001

Table S2. Pearson's correlations among the five sentinel *ABCA7* SNPs (*n* = 634)

	rs3764647	rs3764650	rs115550680	rs3752246	rs4147929
rs3764647	1.000				
rs3764650	0.843***	1.000			
rs115550680	-0.117**	-0.141***	1.000		
rs3752246	-0.139***	-0.004	-0.101*	1.000	
rs4147929	-0.140***	-0.026	-0.110**	0.956***	1.000

* *p*<0.05, ***p*<0.01, ****p*<0.001

Table S3. Association between *ABCA7* sentinel SNPs and general cognitive function (n = 634)

PMID [a]	Ancestry [b]	SNP	Chr	Position	Risk Allele	RAF	Model 1		Model 2		Model 3	
							β_{SNP}	p-value	β_{SNP}	p-value	β_{SNP}	p-value
28480329	AA	rs3764647	19	1044712	G	0.203	-0.04	0.518	-0.02	0.786	-0.01	0.823
21460840	EA	rs3764650	19	1046520	G	0.254	-0.03	0.598	-0.02	0.768	-0.02	0.716
23571587	AA	rs115550680	19	1050420	G	0.060	-0.03	0.748	-0.01	0.928	-0.01	0.884
21460841	EA	rs3752246	19	1056492	G	0.042	0.21	0.088	0.15	0.180	0.15	0.186
24162737	EA	rs4147929	19	1063443	A	0.049	0.21	0.075	0.12	0.243	0.12	0.241
-	-	*APOE* ε2	19	45411941	T	0.122	0.07	0.317	0.11	0.087	-	-
-	-	*APOE* ε4	19	45412079	C	0.226	-0.11	0.046*	-0.12	0.022*	-	-

Abbreviations: PMID, Pubmed ID; AA, African American; EA, European American; Chr, chromosome; RAF, risk allele frequency in GENOA

a. Pubmed ID numbers for studies that identified sentinel SNPs in the *ABCA7* region in association with Alzheimer's disease.

b. Ancestry of cohorts in which significant associations were identified between sentinel SNPs in the *ABCA7* region and Alzheimer's disease

Model 1: General cognitive function ~ SNP + age at cognition measurement + sex + PC1-4 + familial relatedness (random effect)

Model 2: Model 1 + educational attainment

Model 3: Model 2 + *APOE ε2* + *APOE ε4*

*p<0.05; no associations were significant after Bonferroni correction at $\alpha = 0.05/5 = 0.01$.

Table S4. Association of CpGs in the *ABCA7* region and general cognitive function (p<0.05; n = 494)

CpG site	Position	Site Type	Relation to CpG Island	Model 1		Model 2		Model 3	
				β_{CpG}	*p*-value	β_{CpG}	*p*-value	β_{CpG}	*p*-value
cg22271697	1042537	Gene Body	North Shelf	0.08	0.009*	0.07	0.007*	0.08	0.004*
cg00874873	1051161	Gene Body	CG Island	0.12	0.074	0.13	0.025*	0.12	0.034*
cg11714200	1065689	Promoter	North Shore	0.06	0.101	0.08	0.030*	0.07	0.037*
cg26264438	1039942	Promoter	CG Island	0.53	0.236	0.84	0.039*	0.83	0.041*
cg12082025	1064219	Gene Body	CG Island	0.05	0.394	0.11	0.047*	0.11	0.042*
cg18644543	1067356	1st Exon; 5' UTR	CG Island	-0.51	0.031*	-0.33	0.132	-0.34	0.118

Model 1: General cognitive function ~ CpG site + sex + age at cognition measurement + age difference between methylation and cognition measurements + smoking status+ PC1-4 + familial relatedness (random effect)

Model 2: Model 1 + educational attainment

Model 3: Model 2 + *APOE* ε2 + *APOE* ε4

*p<0.05; No associations are significant at FDR q<0.1

Table S5. Interaction between *ABCA7* sentinel SNPs and CpG sites on general cognitive function (p<0.05; n = 494)

SNP * CpG site Interaction	CpG position	Main effects				Interaction	
		β_{SNP}	p-value	β_{CpG}	p-value	$\beta_{interaction}$	p-value
rs3764647 * cg00135882	1065783	-0.01	0.875	0.24	0.086	-0.80	1.46×10^{-4} **
rs115550680 * cg06169110	1046615	-0.23	0.045*	0.06	0.143	-0.38	2.18×10^{-4}**
rs115550680 * cg17316918	1056930	-0.05	0.661	-0.06	0.164	0.41	4.84×10^{-4} **
rs3764647 * cg22271697	1042537	-0.07	0.319	0.16	7.23×10^{-6}*	-0.18	5.77×10^{-4} **
rs115550680 * cg05372495	1063625	-0.04	0.707	4.92×10^{-3}	0.837	0.17	0.008*
rs115550680 * cg02913166	1041178	-0.10	0.329	-0.02	0.244	0.17	0.010*
rs115550680 * cg09467711	1037732	-0.26	0.049*	-0.01	0.632	0.10	0.011*
rs115550680 * cg12817436	1068561	-0.01	0.961	-0.02	0.376	0.20	0.011*
rs115550680 * cg07726048	1039944	-0.03	0.799	0.30	0.031*	-1.02	0.012*
rs115550680 * cg07690733	1066986	-0.11	0.301	-0.14	0.60	2.27	0.014*
rs115550680 * cg07325521	1040062	-0.02	0.872	-0.12	0.627	-1.50	0.015*
rs3764647 * cg09467711	1037732	-0.07	0.315	0.03	0.109	-0.07	0.017*
rs3752246 * cg06169110	1046615	4.94×10^{-3}	0.967	-0.02	0.597	0.24	0.033*

Model 4: General cognitive function ~ SNP + CpG + SNP*CpG + age at cognition measurement + age difference between methylation and cognition measurements + sex + educational attainment + *APOE ε2* + *APOE ε4* + smoking status + PC1-4 + familial relatedness (random effect)

* p<0.05; ** FDR q<0.1

Table S6. Pearson's correlations among *ABCA7* CpG sites[a] (*n* = 494)

	cg00135882	cg22271697	cg06169110	cg17316918	cg00874873	cg11714200	cg26264438	cg12082025	cg18644543
cg00135882	1.000								
cg22271697	0.243***	1.000							
cg06169110	0.273***	0.085	1.000						
cg17316918	0.114*	0.152***	-0.051	1.000					
cg00874873	0.056	0.166***	0.037	0.216***	1.000				
cg11714200	0.128**	0.173***	-0.039	0.213***	0.139**	1.000			
cg26264438	-0.291***	-0.119**	-0.105*	-0.259***	-0.091*	-0.104*	1.000		
cg12082025	0.400***	0.121**	0.217***	0.151***	0.043	0.070	-0.223***	1.000	
cg18644543	-0.220***	-0.130**	0.041	-0.380***	-0.235***	-0.152***	0.407***	-0.125**	1.000

a. CpG sites in this correlation matrix were chosen from Tables 2 and S4. Cg00135882, cg22271697, cg06169110 and cg17316918 are significant CpG sites in the SNP-by-CpG interactions on general cognitive function (FDR q<0.1; Table 2). Cg22271697, cg00874873, cg11714200, cg26264438, cg12082025 and cg18644543 are nominally associated with general cognitive function (p<0.05; Table S4).

* p<0.05, **p<0.01, ***p<0.001

Table S7. Estimated effect of CpG site on general cognitive function for given *ABCA7* SNP genotype group, after excluding outlying values for CpG sites[a]

SNP	CpG site	Genotype	β_{CpG}	*p*-value
rs3764647	cg00135882	AA	0.14	0.311
		GG/AG	-0.49	0.005*
rs3764647	cg22271697	AA	0.14	1.00×10^{-4} *
		GG/AG	-0.02	0.719
rs115550680	cg06169110	AA	0.06	0.130
		GG/AG	-0.37	2.00×10^{-4} *
rs115550680	cg17316918	AA	-0.05	0.238
		GG/AG	0.33	0.004*

a. Outliers greater or less than 4 standard deviations were excluded: 4 values were excluded for cg00135882 (*n* = 490), 2 values were excluded for cg22271697 (*n* = 492) and cg17316918 (*n* = 492), and 1 value was excluded for cg06169110 (*n* = 493)
Model 4: General cognitive function~ SNP + CpG + SNP*CpG + age at cognition measurement + age difference between methylation and cognition measurements + sex + educational status + *APOE* ε2 + *APOE* ε4 + smoking status + PC1-4 + familial relatedness (random effect)
* $p<0.05$

Table S8. Estimated effect of CpG[a] site on general cognitive function for given *ABCA7* SNP genotype group, after adjusting for SNP effect

SNP	CpG site	Genotype	β_{CpG}	*p*-value
rs3764647	cg22271697	AA	0.15	1.00×10^{-4} *
		GG/AG	-0.02	0.571
rs115550680	cg06169110	AA	0.06	0.120
		GG/AG	-0.37	2.00×10^{-4} *

a. Sensitivity analysis was conducted on identified SNP-by-CpG interactions from Table 2 whose CpGs were associated with their corresponding SNPs (P<0.05). The SNP effect was adjusted out of the CpG site effect, and the interaction analysis was conducted using the adjusted CpG value

Model 4: General cognitive function~ SNP + CpG + SNP*CpG + age at cognition measurement + age difference between methylation and cognition measurements + sex + educational status + *APOE* ε2 + *APOE* ε4 + smoking status + PC1-4 + familial relatedness (random effect)
* $p<0.05$

Table S9: Interaction between *ABCA7* sentinel SNPs and CpG sites on neurocognitive measurements (*n* = 494)

DSST	Main effects				Interaction	
	β_{SNP}	*p*-value	β_{CpG}	*p*-value	$\beta_{interaction}$	*p*-value
rs3764647 * cg00135882	-0.35	0.679	0.68	0.709	-7.73	0.005*
rs3764647 * cg22271697	-0.88	0.307	1.22	0.008*	-1.37	0.047*
rs115550680 * cg06169110	-2.19	0.145	0.49	0.340	-4.24	0.002*
rs115550680 * cg17316918	-0.07	0.959	-0.54	0.319	3.38	0.028*

COWA-FAS	Main effects				Interaction	
	β_{SNP}	*p*-value	β_{CpG}	*p*-value	$\beta_{interaction}$	*p*-value
rs3764647 * cg00135882	-0.20	0.828	2.85	0.143	-6.79	0.023*
rs3764647 * cg22271697	-0.64	0.488	1.15	0.021*	-1.04	0.158
rs115550680 * cg06169110	-1.67	0.300	0.01	0.978	-1.76	0.219
rs115550680 * cg17316918	-0.59	0.684	0.07	0.905	3.56	0.030*

RAVLT	Main effects				Interaction	
	β_{SNP}	*p*-value	β_{CpG}	*p*-value	$\beta_{interaction}$	*p*-value
rs3764647 * cg00135882	0.53	0.055	0.19	0.747	-0.87	0.346
rs3764647 * cg22271697	0.50	0.070	0.27	0.075	-0.48	0.036*
rs115550680 * cg06169110	-0.32	0.511	0.07	0.688	-0.71	0.107
rs115550680 * cg17316918	0.09	0.831	-0.06	0.737	1.20	0.017*

SCWT	Main effects				Interaction	
	β_{SNP}	*p*-value	β_{CpG}	*p*-value	$\beta_{interaction}$	*p*-value
rs3764647 * cg00135882	-0.55	0.498	2.92	0.089	-7.68	0.004*
rs3764647 * cg22271697	-1.06	0.187	1.68	1.21×10^{-4} *	-1.79	0.006*
rs115550680 * cg06169110	-2.70	0.058	0.93	0.056	-3.29	0.009*
rs115550680 * cg17316918	-1.23	0.340	-0.89	0.083	3.52	0.016*

TMTA	Main effects				Interaction	
	β_{SNP}	*p*-value	β_{CpG}	*p*-value	$\beta_{interaction}$	*p*-value
rs3764647 * cg00135882	-0.03	0.333	0.05	0.484	-0.23	0.043*
rs3764647 * cg22271697	-0.05	0.143	0.06	0.002*	-0.07	0.020*
rs115550680 * cg06169110	-0.08	0.187	0.02	0.423	-0.15	0.006*
rs115550680 * cg17316918	-0.01	0.903	-0.02	0.272	0.11	0.089

Key: DSST, Digit Symbol Substitution Task; COWA-FAS, Controlled Oral Word Association Test; RAVLT, Rey Auditory Verbal Learning Test; SCWT, Stroop Color-Word Test; TMTA, Trail Making Test A

Model 4: Cognitive test score ~ SNP+ CpG + SNP*CpG + age at cognition measurement + age difference between methylation and cognition measurements + sex + educational attainment + *APOE* ε2 + *APOE* ε4 + smoking status + PC1-4 + familial relatedness (random effect)
* $p<0.05$

Table S10. Interaction between *ABCA7* sentinel SNPs and CpG sites[a] on transcripts in the *ABCA7* gene region (p<0.05; n = 429)

		Main effects				Interaction	
**Transcript**	**SNP * CpG site Interaction**	β_{SNP}	***p*-value**	β_{CpG}	***p*-value**	$\beta_{interaction}$	***p*-value**
ENST00000525939	rs115550680 * cg17316918	0.03	0.428	-9.82×10^{-3}	0.493	0.09	0.026*
ENST00000531467	rs3764647 * cg22271697	0.03	0.085	-0.012	0.270	0.03	0.046*

Model 5: Transcript ~ SNP + CpG + SNP*CpG + age at gene expression measurement + age difference between methylation and gene expression measurements + sex + PC1-4 + familial relatedness (random effect)

a. Significant SNP-by-CpG interactions in Table 2
* p<0.05; No associations are significant at FDR q<0.1

Table S11. Estimated effect of CpG site on *ABCA7* transcripts for given *ABCA7* SNP genotype group (*n* = 429)

Transcript [a]	SNP	CpG site	Genotype	β_{CpG}	*p*-value
ENST00000531467	rs3764647 [b]	cg22271697	AA	-0.01	0.319
			GG/AG	0.02	0.120
ENST00000525939	rs115550680 [c]	cg17316918	AA	-7.6×10^{-3}	0.597
			GG/AG	0.07	0.054

a. Transcripts associated with previously identified SNP-by-CpG interactions in Table S10
b. GG (*n* = 15) and AG (*n* = 156) groups were combined in the GG/AG group (*n* = 151)
c. GG (*n* = 3) and AG (*n* = 47) were combined in the GG/AG group (*n* = 50)

Model 5: Transcript ~ SNP + CpG + SNP*CpG + age at gene expression measurement + age difference between methylation and gene expression measurements + sex + PC1-4 + familial relatedness (random effect)

No associations are significant at $p<0.05$

Table S12. Association of SNPs[a] on transcripts in the *ABCA7* gene region (p<0.05; n = 429)

Transcript	SNP	β_{SNP}	p-value
ENST00000531467	rs115550680	-0.13	3.17×10^{-5}**
ENST00000527496	rs115550680	-0.13	2.14×10^{-4}**
ENST00000529442	rs115550680	-0.10	5.07×10^{-4}**
ENST00000524850	rs115550680	-0.09	0.001**
ENST00000526885	rs115550680	-0.06	0.008**
ENST00000532194	rs115550680	-0.07	0.009**
ENST00000433129	rs115550680	-0.06	0.012**
ENST00000525238	rs115550680	-0.06	0.012**
ENST00000263094	rs115550680	-0.05	0.015**
ENST00000530703	rs115550680	-0.06	0.024**
ENST00000435683	rs115550680	-0.05	0.026**
ENST00000530703	rs3764647	0.03	0.037*

Model 5: Transcript ~ SNP + age at gene expression measurement + sex + PC1-4 + familial relatedness (random effect)

a. SNPs shown were previously significant in the SNP-by-CpG interactions in Table 2

* p<0.05, ** FDR q<0.1

Table S13. Association of CpG sites[a] on transcripts in the *ABCA7* region (p<0.05; n = 429)

Transcript	CpG Site	β_{CpG}	*p*-value
ENST00000531478	cg06169110	0.02	0.008*
ENST00000526885	cg06169110	0.02	0.037*

Model 5: Transcript ~ CpG + age + age difference between methylation measurement and gene expression measurement + sex + PC1-4 + familial relatedness (random effect)

a. CpG sites shown were previously significant in the SNP-by-CpG interactions in Table 2

*p<0.05; No associations are significant at FDR q<0.1
